# Smart pareto-optimized genetic algorithm for energy-efficient clustering and routing in wireless sensor networks

**DOI:** 10.1038/s41598-025-09117-5

**Published:** 2025-10-08

**Authors:** M. Rajalakshmi, S. Ponni Alias Sathya

**Affiliations:** 1 Department of Artificial Intelligence and Data Science, Dr. Mahalingam College of Engineering and Technology, Pollachi, Tamilnadu India; 2 Department of Information Technology, Dr. Mahalingam College of Engineering and Technology, Pollachi, Tamilnadu India

**Keywords:** Sensor networks, Energy efficiency, Genetic algorithm, Clustering, Routing, Load balancing, Network lifespan optimization, Energy science and technology, Engineering, Mathematics and computing

## Abstract

Healthcare, business, and the military employ wireless sensor networks (WSNs). Unfortunately, these networks have power supply, storage, and computing restrictions for sensor nodes. To overcome these difficulties, enhance energy efficiency, and extend network lifetime, we present a novel Pareto-based Genetic Algorithm for Energy-Efficient Clustering and Routing (PGAECR). It incorporates the best results from earlier networking sessions into the starting population for the present rounds, improving convergence speed and solution quality in the search process. The technique combines decisions about clustering and routing into one chromosome. A multi-objective fitness function that takes into account total energy consumption, residual energy balance, load distribution, and network longevity evaluates it. The first group comprises the best-performing solutions from the past, designed to aid convergence and enhance solution quality. An experimental examination examines factors such as transmission energy (ET, ER), data packet length, amplifier energy models (Efs, Emp), communication range, and node density across different network conditions. Experimental results indicate that PGAECR outperforms five other methods, demonstrating superior load balancing with minimal variance in cluster head loads across various scenarios. The proposed algorithm reduced energy usage by 12.4% and increased network longevity by 15.7% compared to conventional clustering and routing methods.

## Introduction

Data transfer between Wireless Sensor Network (WSN) nodes requires multi-hop communication due to energy and transmission range limitations. Until they reach their destination, nodes carry messages directly or indirectly^1^. Since WSNs are fundamental to the Internet of Things, researchers have focused on improving their quality of service (QoS) using multi-objective algorithms^2^. Several multi-objective techniques have been proposed to tackle multiple issues efficiently. This process addresses various issues, including energy consumption, network routing protocols, and load-balancing strategies, to ensure adequate data transfer and meet high-performance standards^3^. Proactive routing in table-driven networking maintains endpoint routes regardless of requirement. Thus, path knowledge is readily available when a receiver’s route is needed^4^. This proactive grouping will waste energy sending the latest facts without data transmission. Reactive or on-demand routing reduces route overhead by eliminating route management when no information is transferred^5^. Overflow traffic requires excessive energy, which reduces network efficiency and quality of service. Reactive methods seek alternatives. Link failure adds to the network’s stress and consumes energy during route-finding^6^. These will increase load, reduce throughput, and increase packet delivery, decreasing network efficiency. Building an energy-efficient, high-performance system is crucial. A detailed study focused on mobile node energy efficiency to extend network lifespan^7^.

A well-known routing technique called ad-hoc on-demand multi-path distance vectors (AOMDV) chooses paths with the fewest possible hops. Without the requirement for route exploration, AOMDV offers alternative paths in the event of node failure or channel separation, thereby reducing latency and maximizing throughput^8^. Conversely, suppose an element or link failure causes the data to be broken for a single path-forwarding technique. In that case, data transmission will be terminated, and the task of identifying a new path should be initiated, resulting in a decrease in network efficiency^9^. To put it another way, a node may experience a battery failure during information transfer, causing it to interrupt and necessitate a switch to an alternative accessible path. Several research studies have examined Energy-saving routing techniques^10^, with a primary focus on cost. In^11^, the approach was more sophisticated, requiring more processing time but less power. On the other hand, these procedures initiated an exciting discovery in the event of a route loss.

However, energy, storage, and computational overhead limit these networks’ performance and lifespan. Unfortunately, existing routing and clustering algorithms don’t always balance energy usage, causing sensor nodes to burn out fast and decreasing network efficiency^12^. Traditional approaches fail to react to network performance data, resulting in suboptimal routing and clustering decisions. Adaptive clustering and routing must be used to extend network life and reduce energy use immediately^13^.

In this work, the proposed PGAECR model contributes in many ways, enhancing energy efficiency by optimizing clustering and routing, folding in optimal solutions of previous rounds, and injecting load balancing into the fitness function. Energy consumption is minimized while the operational lifetime of the network is maximized. Besides this, PGAECR improves the efficiency of the search by rapidly converging to high-quality solutions and reducing computational overhead. The model effectively handles load balancing to maintain an equal energy distribution at nodes and avoid a local energy drain. The revolutionary element of PGAECR is that it may incorporate the best-performing solutions from prior networking sessions in the starting population for the current optimization cycle. This strategy enhances search efficiency and convergence more than standard genetic algorithms that start with random solutions^14^. Our technique integrates routing and clustering decisions into a single chromosome, ensuring an integrated optimization process. The fitness function’s load-balancing parameters ensure a consistent node energy distribution, thereby reducing the likelihood of early sensor failure^15^. Experimental results demonstrate that PGAECR outperforms existing methods in terms of energy efficiency, exhibiting the least variation in cluster head loads and the lowest average energy consumption. Its architecture allows scaling and flexibility; hence, it becomes highly applicable in large-scale networks and diverse network deployments, proving its robustness in dynamic and complex environments. The significant contributions of the proposed model are:

PGAECR embeds route and grouping choices into a single chromosomal to avoid complications in optimizing.

The uniqueness of PGAECR lies in its ability to incorporate the optimum resolution from the preceding iteration of the network into the starting population of the current cycle. Thus, energy conservation and appropriate load balancing among nodes are ensured by the direct relationship between the health function and overall energy use.

The experimental findings reveal that PGAECR has the most outstanding load distribution and lowest head cluster load volatility. In addition, when compared to other approaches, it achieves the maximum energy performance and the lowest average power consumption of each node and head in the cluster.

Forresolving the multi-objective issues with few resources such as Wireless Sensor Networks (WSNs), Pareto optimization is the best. It attempts to pick the optimum for performing many conflicting ones: to store as few energies as possible and network longevity has to be optimized. This is compared to single-objective or weighted-sum answers that in some specific areas skip important trade-offs. Alternatively, this made the achievement in some balanced overseveral parameters. Therefore, Pareto-based Genetic Algorithm (PGAECR) is selected because it is capable of justifying increased-quality solutions effectively with the help of historically optimum answers among new populations. The method fastens the search process, increasing clustering and routing efficiency and flexibility. These have led to enhanced load balancing, reduced energy utilization as well as the improved scalability in different WSN environments. 

Optimal system performance, resource allocation as well as communication technique have increasingly determined by the proper routing and clustering. There is a indeed for the proper methodologies to enhance the efficient methodology in many real-time utilizes like transportation methodologies, logistics and WSNs. The authors had given the unique hybrid optimization technique which enhances decision-making by adding routing along with clustering systems in a one chromosome. The method focuses to optimize the performance of system with low computing complexity by the joining of several methods.

## Related works

This section discusses related works on optimizing WSNs using various strategies. To solve the problem of precisely identifying nodes containing wireless sensors in a roaming network setting, a new localization technique project is being developed for mobile sensor networks^16^.

Environment and other dependent issues are in vogue now. An appropriate node sensing point was essential to facilitate the enough information processing, resource sharing and network management in these networks. In mobile sensor networks, traditional localization operations are related to the angle of arrival, transferred signal strength, departure time and time difference arrival. The cons of these methods was complexity, low accuracy and hardware dependence^17^. Consequently, it is necessary to develop innovative localization techniques that can surpass these limitations and provide node-based localization that is more precise and effective. This work employed optimization and machine learning approaches to develop and apply a novel localization method. The suggested method^18^ delivers accurate localization in various network settings while addressing the shortcomings of current methodologies.

Additionally, this work employs simulation and empirical experiments to assess and compare the proposed approach with existing methods. The goal of developing a new localization method for wireless networked sensors is to enhance the effectiveness of various applications in this sector by creating a more accurate and efficient localization technique. AROS, a meta-heuristic algorithm modeled by rabbit survival strategies, was introduced in^19^. The effectiveness of ARO was assessed by comparing its results with those of other optimization methods on five engineering issues and 31 benchmark functions. The results showed that ARO consistently outperforms its competitors in handling these challenges. Moreover, the real-world implementation of the ARO optimizer in rolling bearing defect diagnostics demonstrates the optimizer’s efficacy in addressing intricate problems. The study presented a unique swarm intelligence method called NGOs that mimics the hunting style of northern goshawks^20^. The algorithm’s performance was compared to eight widely used algorithms and assessed across 68 function objectives. Simulation outcomes showed that the NGO performed better than expected, further supported by the fact that it solved four real-world design challenges. The research utilized ten jumbled maps from The Hunger Games Searching (HGS) approach^21^, focusing on animals’ foraging habits and hunger senses. HGS performs better in three situations when chaotic maps are included, with the second one showing quicker convergence. The technique’s effectiveness was assessed utilizing CEC2017, 23 traditional benchmark issues, and real-world technical tasks; the findings showed promise compared to previous research. A population-based metaheuristic method, known as the Slime Mould Algorithm (SMA)^22^, strikes a compromise between exploration and exploitation. An improved version, known as the MSMA, was presented in a research study. It employs a spiral search methodology, adaptive parameter management approaches, and chaotic opposition-based learning. Compared to other computations, the MSMA performed better in terms of convergence precision, speed, and security in real-world applications.

The new protocol, ReLeC-MO, developed by Regilan et al.^23^, combines the ReLeC clustering algorithm with multi-objective optimization to meet the increasing need for energy-efficient WSNs in the Internet of Things (IoT) ecosystem. Optimizing network design for energy efficiency is achieved by ReLeC using reinforcement learning-based clustering. In our comprehensive simulations, ReLeC-MO achieves better results than previous methods. Reduced latency by 39%, energy consumption by 50%, and throughput by 25% are all claims made by ReLeC-MO. With a 20% increase, the network outlasts the most recent model. Written in MATLAB, it’s easy to replicate and modify for other IoT applications.

Li et al.^24^ propose near-Pareto multi-objective routing optimization for SASIN (space-air-sea integrated network) using MOCOP to balance several goals. The SASIN system model uses channel models of ship, aircraft, and satellite communication links. We utilize multi-objective evolutionary algorithms to approximate Pareto-optimal solutions. Two improved non-dominated sorting genetic algorithms, INSGA-2 and ISPEA-2, can approximate the Pareto optimal set. The simulation results suggest tackling the multi-objective routing problem can yield reasonable trade-off solutions for flexible communication link selection.

Harizan et al.^25^ propose many evolutionary algorithms (EAs) to help build Relay Nodes while balancing aims. Solution vectors were well encoded. For the assessment of solution vectors, all the objective functions were gained efficiently. Every prescribed method were basically formulated. The superior method was demonstrated for the issue by recognizing the facts. Simulations denoted that MODE had been compared along with the other dependent methodologies for the given title. Later, Post hoc LSD testing had been accompanied with the performance of ANOVA. 

Through the utilization^26^ of moveable sinks in IoT-enabled HWSNs (OptiGeA), a genetic algorithm-based data monitoring and regulating approach utilizing IoT-enabled WSNs is provided. To enhance the OptiGeA method for CH elections modified the fitness function to incorporate density, distance, energy with heterogeneous node capacity. The working on OptiGeA had been comprised of a sink, numerous static sinks and many moving sinks. There presents an unproperly biased test. While adding the DDC mechanism, the OptiGeA-DDC scheme is better than the MS-GAOC scheme by 48.33%. Subsequently, the OptiGeA scheme made better performance over GAOC by 10.44%. 

The author^27^ presented an innovative approach for efficient clustering that combines the greylag goose method with other methods. The accuracy of the golden sine technique along with the effectiveness of greylag goose optimization had been incorporated here. Within 6G-powered IoMT networks, the methodology exploited the Lévy flight mechanism for chosing the optimal cluster head. Blockchain technology had been utilized in the securing data and openness enhancement.

A whale optimization technique^28^ and a harmony search method are created to find the intermediate and cluster head nodes needed for routing, respectively, using the NP-Hard clustering format. NS-3 simulator confirmed that the presented approach had been superior to conventional one in every aspects like longlasting, energy usage and active nodes through idle nodes. The given methodology had shown more efficacy over the other conventional clustering techniques accompanied with the consumption of low power in the system.

The Internet of Things is very important in many fields^29^, such as health care and medical systems. In rare situations, this device sends information, including a patient’s heart rate, blood pressure, oxygen saturation, and temperature.Low-power nodes had been interconnected with a patient’s body to the healthcare center which communicated often. If the nodes drew power unequally, it seems improper to transfer data to the data centers. For the devices to be capable to convey with one other in a cost-efficientway, a supportive routing system had to be there. It had permitted for optimal functioning whenever there occurs the energy saving. Aoptimal methods of power savings and performing the system’s duration was clustering as a routing methodology. This recent paper had given a black widow optimization method for the optimal routing-based intermediate and the picking of cluster head node with NP-Hardness of clustering and also a harmony search method.

The author introduced GECR^30^, a genetic algorithm-based method for energy-efficient clustering and routing. The authors looked for the optimal solution by integrating the outbest result from the end round of networks over the commencing population in upgoing round. The cluster and routing algorithms were placed on the same chromosome to observe the quantity of energy consumed. These reduced the system’s energy cost by expressing the fitness function as a function of energy cost. Load balancing when constructing the fitness function was also considered. 

The author suggested^31^ an energy-efficient method for choosing the best number of cluster heads and grid heads (EOCGS) that will make the network last longer. In this section, they first provided the formula for the best number of clusters. Then, they offered a novel method for selecting the optimal number of CHs that saves energy. To conserve the energy of the CHs, the Grid Head (GH) concept is being implemented in a dynamic mode. When there are more CHs than the threshold limit, some of them work as GHs. The suggested fitness function, which is based on the residual energy, Euclidean distances, and the position of the grid centroid of the CHs, is used to choose these CHs.

The author presented^32^ a section by using the formula for the optimal number of clusters. Then, a unique strategy had been given over the determination of the CH configuration that consumes minimal energy. The undefined version of the Grid Head (GH) methodology was exploited in an attempt for reducing the power usage in CHs. Some of the CHs protruded as GHs in the case of excessiveness. The given fitness function had picked those CHs dependent to the lefover energy, Euclidean distance in-between and gridcentroid CHs locating. The author had given a novel WSN ordered mechanism named Energy Efficient Hybrid Clustering and Hierarchical Routing (EEHCHR). This had been a new method that exploited hybrid and adaptive clustering, base station locating, node residual energy, the Fuzzy C-Means algorithm and Euclidean distance parameter for storing the power.

In^33^, the network had seemed power-saving since the clustering was not present in the respected situation. Later, the fitness algorithm had took each CH. It improved the CH selection process based on the energy of nodes present in a scenario-dependent fashion. Two additional CHs, DCH and CCH chosen by different fitness functions had been enough. LP/NLP and PSO were provided as the possibility techniques for those scenarios. The routing method was presented with a fitness function that generalized over the varied range of utility and an proper functioning of encoding scheme for pieces. Clustering was the title that the nodes stored power by the means of even load.

In^34^, few experiments had been carried out on the algorithms as proposed, and these were contrasted against the existing methodologies. The results demonstrated that those acquainted better performance than others due to the reduced energy utility, forwarded several data packets to the base station, assembled a few dead sensor nodes and kept it a longlasting overall network life duration. The author has provided two new clustering methods, HOCK and HECK that used low energy and made the network last long in homogeneous and heterogeneous settings. These techniques had strong reliance on herd search algorithms including Krill and Cuckoo. Krill herd method assisted in obtaining the optimal cluster centers and cuckoo search assisted in obtaining the optimal nodes. The performance of HOCK algorithm was validated through varying nodes and base station location. 

A WSN has to employ communication networks that use less energy. Some of the energy limitations of WSNs include clustering, storage, communication capacity, high configuration complexity, poor communication speed, and limited computing capabilities^35^. Additionally, selecting a cluster head remains a challenge for energy reduction in WSNs. In this study, the Adaptive Sailfish Optimization (ASFO) method, combined with K-medoids, is employed to cluster sensor nodes (SNs) together. The primary objective of the research is to enhance the selection of cluster heads by reducing the distance, stabilizing energy consumption, and minimizing latency between nodes. An energy-efficient cross-layer-based expedited routing protocol (E-CERP) is employed to determine the most efficient route while minimizing network overhead in real-time. Table 1 shows the Comparative Summary of Existing Strategies in WSN Optimization.

**Table 1:** Comparative summary of existing strategies in WSN optimization.

Ref.	Methodology	Advantages	Disadvantages
^16^	Variational Autoencoders for blind equalization	Improves signal decoding accuracy in noisy environments	High computational complexity, not tailored to WSNs
^17^	Hybrid Gorilla Troops + Gradient-based Optimizer	Effective for tuning system stabilizers	Limited to power systems, lacks adaptability to WSN
^18^	Deep Transfer Learning for seismic detection	Robust accuracy in feature extraction	Requires large labeled datasets
^19^	Lightweight DNN (CAM-FoC) for surgical tools	High-precision, low-latency measurements	Domain-specific; not easily generalizable to routing
^20^	Improved Harris Hawks Optimization	Enhanced exploration and convergence	Sensitive to initial parameters
^21^	AGTO for social welfare bidding	Optimal performance in energy markets	Complex adaptation needed for WSNs
^22^	Quantum AGTO	Robust against local optima	The high time complexity for large networks
^23^	ReLeC-MO (Reinforcement + Multi-objective)	Reduces energy by 50%, improves lifetime	Requires heavy computational setup
^24^	Near-Pareto routing for SASIN	Balanced multi-objective optimization	System-specific assumptions (air/sea/space)
^25^	Evolutionary relay placement	High coverage and connectivity	Convergence time increases with node density
^26^	OptiGeA: GA-based data regulation in IoT	Integrates node capacity and density	Needs multiple moving sinks for peak performance
^27^	Blockchain for secure IoMT routing	Adds security and transparency	Overhead due to consensus protocols
^28^	Hybrid Metaheuristic for pandemic IoT	Combines strengths of multiple algorithms	May suffer from parameter tuning sensitivity
^29^	Black Widow + Harmony for routing	Strong cluster head selection	Complexity increases with scalability
^30^	GA for clustering and routing	Effective in energy saving	Slower convergence in large-scale WSNs
^31^	EOCGS: Grid-head + cluster-head hybrid	Optimized selection improves energy use	Complex grid setup may reduce adaptability
^32^	EEHCHR: Hybrid clustering + hierarchical routing	Improved energy balance and latency	It may require centralized control
^33^	PSO-based clustering and routing	Efficient in dynamic environments	Suffers from premature convergence
^34^	Hybrid PSO-GA for energy-aware routing	Combines global and local search strengths	Increased computational load
^35^	ASFO + Cross-layer routing	Achieves efficient path selection	Cross-layer design is harder to maintain

Methods for optimizing routing and clustering have been the subject of numerous studies. Heuristic and deterministic algorithms, such as shortest-path techniques and hierarchical clustering, are essential to traditional methodologies. Scalability and flexibility to changing contexts are generally challenges for these systems. Adaptable and efficient metaheuristic algorithms, such as Genetic Algorithms (GA), Particle Swarm Optimization (PSO), and Ant Colony Optimization (ACO), are currently the focus of study. In contrast to current approaches, the suggested technique combines routing and clustering into a single framework, guaranteeing a more unified and optimal outcome.

## System methodology

### Overview of proposed model

**Fig. 1:**
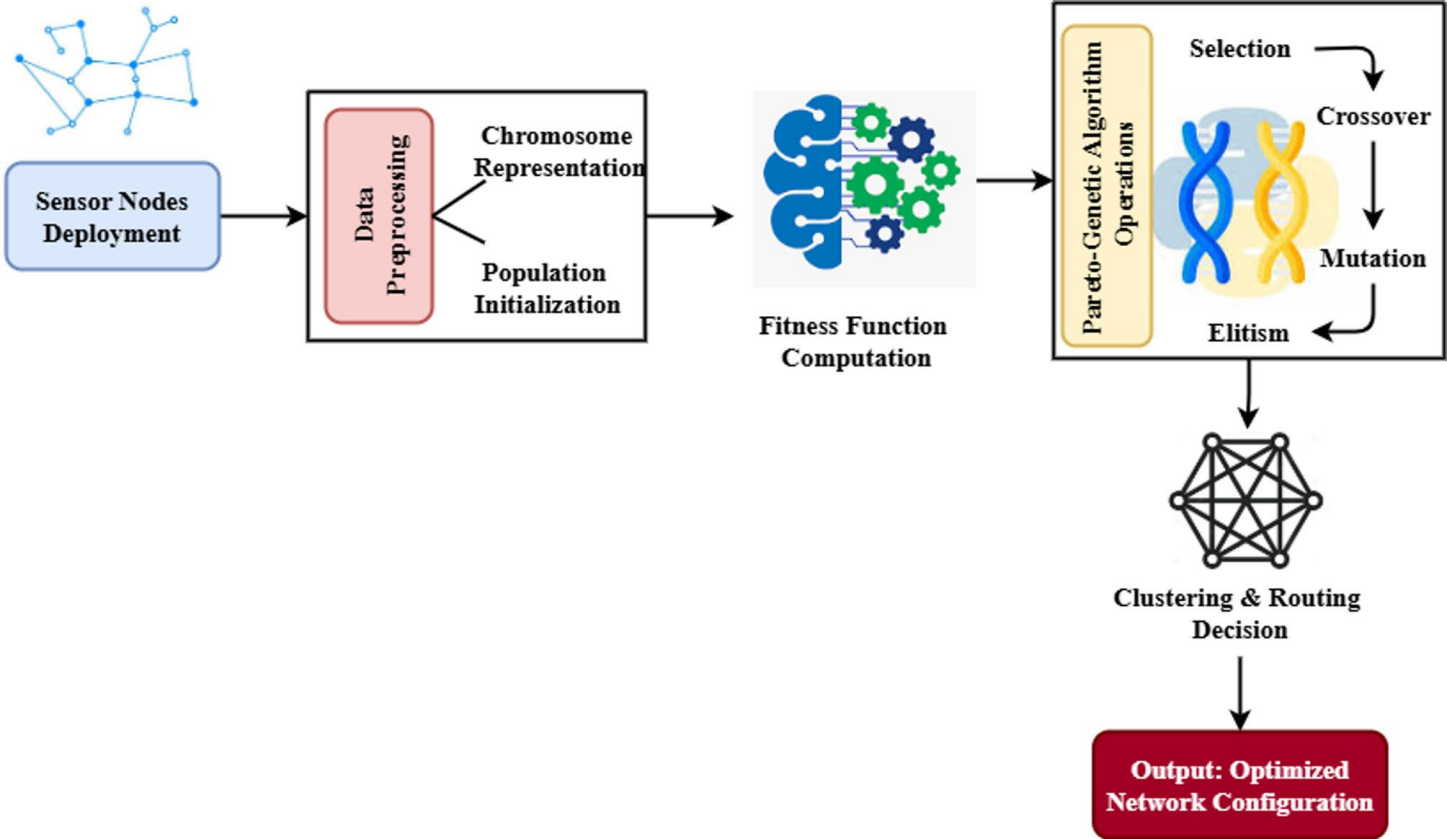
Structure of the proposed model.

An energy-efficient clustering and routing method called PGAECR, which is based on Pareto, is shown in Fig. 1. This method maximizes the energy efficiency and lifespan of WSNs by optimizing their routing and clustering. A Pareto-based Genetic Algorithm (PGA) is an evolutionary optimization approach that combines GA with concepts of Pareto-based multi-objective optimization. In multi-objective problems, when conflicting goals are optimized simultaneously, it thrives. The PGAECR model employs Pareto-based multi-objective optimization to route and cluster WSNs in a novel, energy-efficient manner. The model significantly enhances network longevity and reliability by leveraging historical data and promoting balanced energy use compared to current methods.

Here, research terminology is explained and then, the network design and energy modeling of the proposed PGAECR algorithms are presented. Pareto-based Genetic Algorithm for PGAECR seems to be properly-maintained way as a technique for WSN routing and clustering optimization. The introducing part of this process was the deployment of sensor nodes. These are given either appropriately or inappropriately to the formation of network architecture. A perfect solution needs an effective clustering and routing methodology gave the restricted energy sources for the nodes. As per following, data preparation presented, where the chromatic indication was pointed and the introducing population for the solutions was given. For guaranteeing a maximum convergence rate and search capacity, PGAECR utilized the historical best answers within the initial population than the previous methodologies. This ensured that the method started with a quality candidate solution leading to the optimization outcomes further.

 Regarding the upcoming pre-processing stage estimation of the fitness function, the presented solutions had been given in energy usage, load balancing and network lifetime increase. The Pareto-based Genetic method operations like selection, mutation as well as elitism make the optimization to its core. The genetic operations increase the optimal solutions with several hereditary generations as well as persistently increasing the population. The utmost optimized solution gives the clustering and routing methods such as the energy-efficient cluster heads choice and setting up of optimal routing channels. The ultimate outbest is an optimally balanced network construction that enhances the efficiency of WSN, increases network longevity as well as decreases energy utility per node. Table 2 shows the summary of notations.

**Table 2:** Summary of notations.

Notation	Description
$\:N$	Total number of sensor nodes in the network (excluding the sink)
$\:H$	Set of all Cluster Head (CH) nodes
$\:M$	Set of all Cluster Member (CM) nodes
$\:{h}_{j}$	A specific Cluster Head node
$\:{m}_{i}$	A specific Cluster Member node
$\:S$	Sink node or base station
$\:{d}_{ij}$	Euclidean distance between nodes ii and jj
$\:{E}_{T}$	Energy required to transmit 1-bit of data
$\:{E}_{R}$	Energy required to receive 1-bit of data
$\:{E}_{D}$	Data aggregation energy cost per bit
$\:{\epsilon\:}_{fs}$	Amplifier energy in free-space model
$\:{\epsilon\:}_{mp}$	Amplifier energy in multipath model
$\:{d}_{o}$	Threshold distance to switch between free-space and multipath models
$\:l$	Length of the data packet in bits
$\:{E}_{CM}\to\:CH$	Energy used for transmission from CM to CH
$\:{E}_{CH}\to\:S$	Energy used for CH to transmit data to sink (directly or via hops)
$\:{E}_{total}$	Total energy consumption in one communication round
$\:{E}_{hi}$	Average residual energy per load at CH node hih_i
$\:\mu\:$	Mean of average residual energies across all CHs
$\:\sigma\:$	Standard deviation of residual energies (used for load balancing)
$\:F$	Fitness function combining energy consumption and load balancing
$\:Scheme$	Chromosome encoding of routing and clustering decisions
$\:flag$	Initialization condition flag (determines reuse of previous best solution)
$\:nP$	Size of the population in the Genetic Algorithm

### Problem formulation

Clustering and routing methods divide the network’s operation time into rounds, minimizing energy usage during each round to enhance energy efficiency. This is achieved by defining a Boolean value called $\:{c}_{ij,}$ Which is determined using Eq. (1).

1 $$\:{c}_{ij}=\left\{\begin{array}{c}1,if\:{m}_{i}is\:assigned\:to\:{h}_{j}dfsddf\:\forall\:i.j:1\le\:i\le\:nM,1\le\:i\le\:nH\\\:0,otherwise\end{array}\right. .$$

According to Eq. (2), the total energy used in each cycle is comprised of $\:clusteringE$ and $\:routingE$. The connection power used among each membership and their respective CH_S_ is called $\:clustering$ heads.

2 $$\:Clustering\:E=\sum\:_{i=1}^{nM}\sum\:_{j=1}^{nH`}mh{E}_{ij}*{c}_{ij} .$$

The amount of energy used to transfer information from each CH to the node that sinks is called $\:routingE$ represented in Eq. (3)

3 $$\:RoutingE=\sum\:_{i=1}^{nH}{E}_{hihnH+1}\: .$$

Next, the following is the optimum goal for the route and cluster issues is shown in Eq. (4)

4 $$\:\sum\:_{j=1}^{nH}d\left({m}_{i},{h}_{j}\right)*{c}_{ij}<{d}_{max},1\le\:i\le\:nM,{m}_{i}\in\:M,{h}_{j}\in\:H.$$

Any CM network may only connect one CH node ($\:{h}_{j}$). Equation (4) requires that the gap between mi and hj be within mi’s maximum range of communications.

### Sensor deployment nodes

 Under flat topology networks, nodes either conversestraight or through the hops to sinking node. The topology seems flat, stableas well as fundamental. Every detector has to update the routing tables systematically regarding the topology and scalability problems. Whenever there seems to be a lack in administration of any kind like CH, flat-designed nodes expel energy. There present the various sensors on nodes among the organizational systems. There have some CMs and sometimes one CH within every cluster. Once the CH has given its cluster and gathered data from CMs, it is sent to the next-hop node. The second cluster nodes 3, 4 and 2 send the information to CH 2, which accumulates the information and sends the message to CH 1. In Cluster 1,node 2, 3, and 4 contains the gathered information before being sent to the sink node via first CH node. Hierarchical topology possesses a number of advantages: 1) CMs only need to communicate with the CHs; 2) CHs save energy by removing the unnecessary or erroneous data. 

The energy required to convey a l-bit message from network node i to node j is shown in Eq. (5).

5 $$\:{E}_{rx}\left(l,{d}_{i\:j}\right)={E}_{etec}*l+{E}_{amp}\left({d}_{i\:j}\right)*l=\:\left\{\begin{array}{c}{\varepsilon}_{etec}*l+{\epsilon\:}_{fs}*l*{d}_{i\:j}^{2}\:if\:{d}_{i\:j}<\:{d}_{0\:}\:\:\:\\\:{\varepsilon}_{etec}*l+\:{\epsilon\:}_{emp}*l*{d}_{i\:j}^{4}\:if\:{d}_{i\:j}\:\:<\:\:{d}_{0}\end{array}\right. .$$

where $\:{E}_{etec}$ is the power needed to drive and control technological parts:$\:{\:E}_{amp}\:\left({d}_{i\:j}\right)$ is the power used for amplifiers of signals during the transmission of 1-bit information; $\:{\epsilon\:}_{fs}$ and $\:{\epsilon\:}_{mp}$ are the parameters for the open space simulation and multiple paths model, and $\:{d}_{0}$ is the distance limit, which is determined as Eq. (6)

6 $$\:{d}_{0\:}=\:\sqrt{\begin{array}{c}\:\\\:{\epsilon\:}_{fs}/{\epsilon\:}_{\begin{array}{c}mp\end{array}}\end{array}} .$$

Equation (7) states that the Euclidean distance among nodes i and j, d, determines D$\:T\:(l,\:{d}_{ij})$. The portable signal propagates according to the free place models, and the spread energy corresponds to the squared distance $\:{d}_{ij}$ if it is less than the thresholds $\:{d}_{0}$. The separation among vertices i and j, assuming their coordinates are $\:(xi,\:yi)$ and $\:(xj,\:yj)$, respectively.

7 $$\:{d}_{ij}=\:\sqrt{({x}_{i}-\:{x}_{j}{)}^{2}+({y}_{i}-\:{y}_{j}{)}^{2}} .$$

#### Data pre-processing

Data preparation—establishing the chromosomal representation and solution population—improves the optimization process in the PGAECR model. Traditional approaches employ random solutions, whereas PGAECR leverages the best initial population responses from prior network cycles. This gives a better starting point than random solutions used in previous approaches. Convergence velocity and search efficiency improve dramatically. The fitness function is calculated using network energy usage, load balancing, and lifetime optimization. A well-balanced, energy-efficient WSN design starts with pre-processing. This is done by merging past best solutions and improving candidate solutions via selection, mutation, and crossover. This preparation service assures optimization begins with well-organized data. This stage includes population and chromosomal representation. The chromosomal representation encodes grouping and routing solutions, optimizing them concurrently. By incorporating the most successful replies from previous network sessions into the starting population, the PGAECR algorithm enhances efficiency. This differs from random initialization. This historical learning technique accelerates convergence, reduces wasteful computing, and improves solution quality. The energy status, network properties, and node connections are also evaluated during the pre-processing stage. This ensures that only viable and efficient first solutions advance to fitness evaluation. The population initializing technique and the fitness metric were presented, and mutation and crossover operators were discussed.

### Chromosome representation

The IDs of the nodes should be verified and modified before the chromosomal design. Equation (8) shows the updated CH node identification as $\:{h}_{i}.$

8 $$\:ID\left({h}_{i}\right)=\left\{\begin{array}{c}\:\:\:\:i,If\:i\le\:nH\\\:n*i=nH+1\end{array}\right. .$$

where $\:\varvec{i}$ is the CH nodes’ initial identification. Node h_*i*_ new identity is n if it is a source node $\:i=(nH+1)$ else, it stays the same if it is an ordinary CH $\:\left(i<nH\right)$. The routing strategy and the grouping, referred to as a scheme, are encoded on the same chromosomal in the suggested PGAECR method. A scheme’s genome length is equal to the number of all sensor nodes minus the sink node, or n-1. There are two components to the chromosomal system. The route plan and $\:k\le\:nH$ make up the first section. The CH node’s subsequent hop has two alternatives, as shown by Eq. (9):

9 $$\:nextHop\_h{k}_{=\left\{\begin{array}{c}{h}_{jaaa}\:if\:scheme\:\left[j\right]=j,k\le\:nH,j\le\:nH\\\:{h}_{nH+1}\:\:\:\:\:\:if\:scheme\left[k\right]=n,k\le\:nH\end{array}\right.} .$$

The next-hop nodes of the header node $\:k$ are *j* if the k-th chromosome is j, and both k and j are less than or equivalent to $\:nH$.On the other hand, the sinking node $\:nH:1$ is the following-hop node of $\:k$. The grouping design and $\:nH<k<n\:$comprise the second half of the chromosomal design. The component of the node $\:mk$;‘s CH node is computed as follows, shown in Eq. (10).

10 $$\:C{H}_{{m}_{k-nH}}={h}_{j},if\:scheme\left[k\right]=j,nH<k<n,j\le\:nH .$$

The CM node $\:mk$; with identification, k is allocated to the CH node $\:j$ if the $\:k-th\:$gene is $\:j\:$and j is smaller or equivalent to $\:nH$. The internal numbers of the node correspond to sensor cluster identification. Solid arrows represent transmission from CM networks to the respective head nodes, whereas dashed arrows represent routes among CH nodes. The 17 sensor networks in the WSN comprise one sink node, four CH nodes (*H* = *h_1_, h_2_, h_3_, h_4_), and twelve CM nodes (M = *$\:{m}_{1}$, $\:{m}_{2}$, …,$\:{m}_{12}$).

**Fig. 2:**
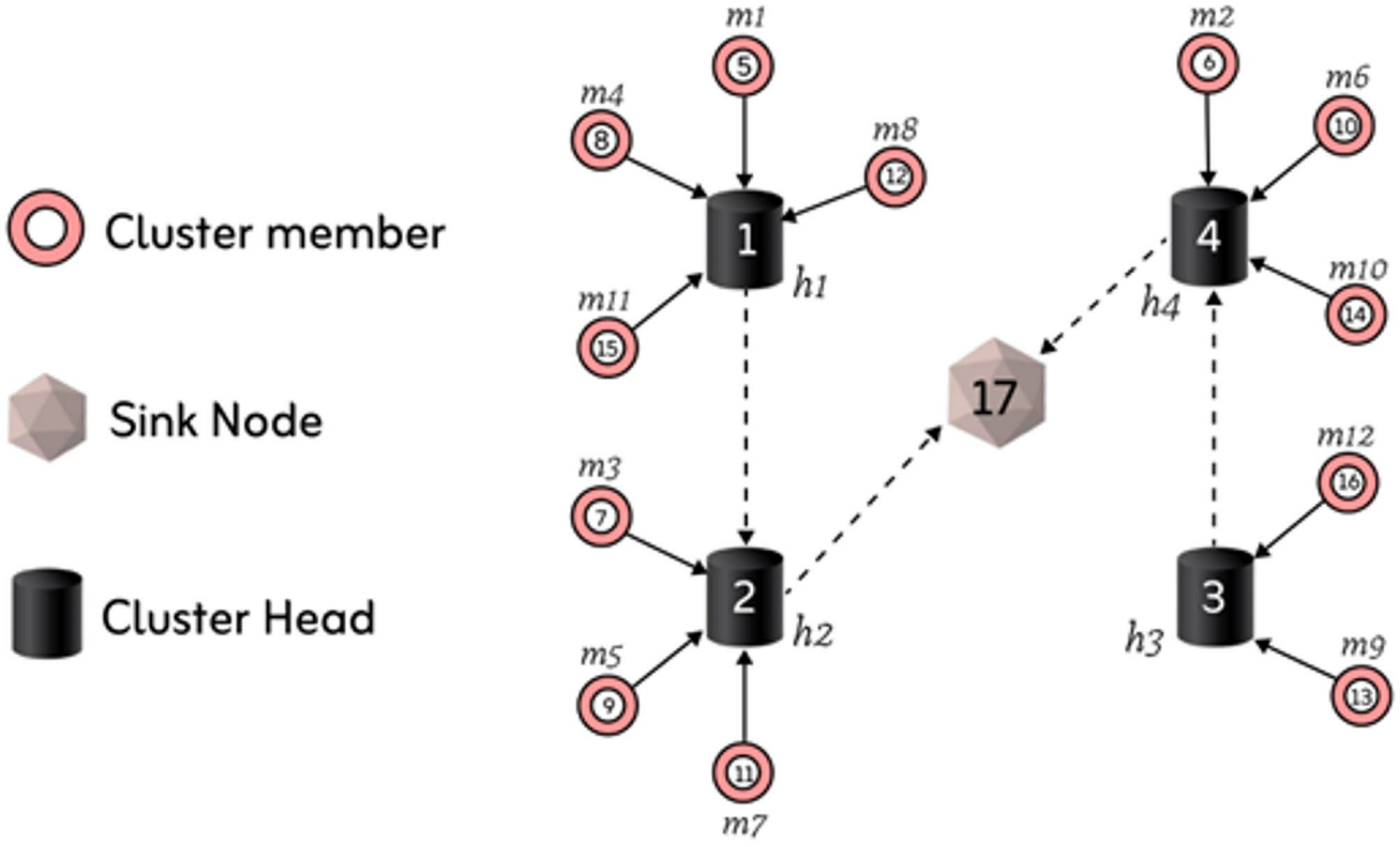
Illustration of proposed WSN route and cluster system.

**Fig. 3:**
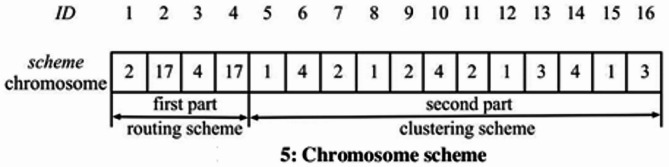
Chromosome scheme.

Figure 3 shows the appropriate chromosomal design for the routing and grouping strategy in Fig. 2. The values in the “ID” row of Fig. 3 represent the sensor nodes’ IDs concerning the appropriate gene location. To optimize data transmission and energy efficiency, Fig. 2 conserves data transmission and power by utilizing a network organization via hierarchical clustering. Sensor cluster nodes pass data to head cluster nodes. Then, the heads consolidate the data and forward it to the sink cluster node. Structured communication renders networks scalable, low in energy usage and avoids redundant transmission. The method is ideal for WSNs or IoT-based systems on a mass level (Fig. 3). An essential part of the suggested approach is the provided chromosomal representation, which encodes routing and clustering techniques in a single structure. Data transfer, communication, and transportation all benefit from the routing strategy, which is represented by the first portion of the chromosome. This ensures that the system’s routing decisions are efficient and effective. Clustering, which comprises the second side of the chromosome, is in charge of grouping components into suitable clusters, which in turn optimizes the distribution of resources and the efficiency of the network. Iteratively improving solutions is achieved by merging both strategies and using evolutionary algorithms or optimization techniques. The chromosome enhances the system’s performance by undergoing fitness evaluation, selection, crossover, and mutation processes to identify the optimal routing and clustering solutions.

The genetic scheme includes the routing and clustering systems. The gene locations stated in the first phase let us identify the CHS in the network. Alleles at gene loci dictate which CH leaps next. To show the identity 17 (sink node h5), the following hop of the CH (identity: 2), corresponding to CH h2 in Fig. 3, is marked by the allele of the gene in the second position (17). The allele of the gene in the third location in the scheme, 4, suggests nodes whose identity is 4, which corresponds to CH h3. In the final position component, gene placements reveal network CMs. The significance of an allele at a gene locus influences CH identification among related populations of origin. For example, Eqs. (11) and (12) produce the CM (identity: 7), which is CM m3, CH h2, where gene position 7 is 2.

11 $$\:scheme\left[k\right]\in\:nextHops\left({h}_{k}\right),if\:k\le\:nH$$

12 $$\:scheme\left[k\right]\in\:pCH\left({m}_{k-nH}\right),ifnH<k<n .$$

### Population initialization

There are many arbitrarily created schematic chromosomes in the starting population. Given the restricted connectivity of sensor nodes, every system chromosome must be verified as genuine. All of the genes for the first segment of the chromosomal sequence need to meet by the Eq. (13):

13 $$\:Scheme\left[k\right]\in\:nexthops\left({h}_{k}\right)ifk\le\:nH .$$

In this case, the k-th CH s value must be included in collecting possible next-hop nodes. Additionally, the value of the k-th gene needs to be included in the list of possible CHs for CM K: K if the gene is found in the second half of the chromosomal structure. The suggested PGAECR approach determines each network round’s best clustering and routing architecture. Every network round has a different optimum scheme due to sensor node energy variations. Equation (14) demonstrates how the optimization goal correlates with energy usage based on node distance. With the predetermined locations of each sensor node in the system, the best route and clustering strategy for the $\:r+1$th connection round is similar to that of the r-th network circle. We want to decrease the amount of GA repeats.

Algorithm 1 depicts population initialization processes. The optimum system from the prior cycle is previous_scheme in pseudocode 1. Note that (1) the system is in the first cycle, and (2) it has one or more broken networks from the prior cycle. In both cases, previous_scheme shouldn’t be part of the initial population. These two circumstances are symbolized by the parameter flag (flag = 0) in Algorithm 1. H denotes a collection of all nodes in the CH other than the sink node, and M represents the set of all CM nodes. Before the network starts up, H and M are set up. The algorithm’s result is the system’s first sampling, which comprises each Scheme chromosome.


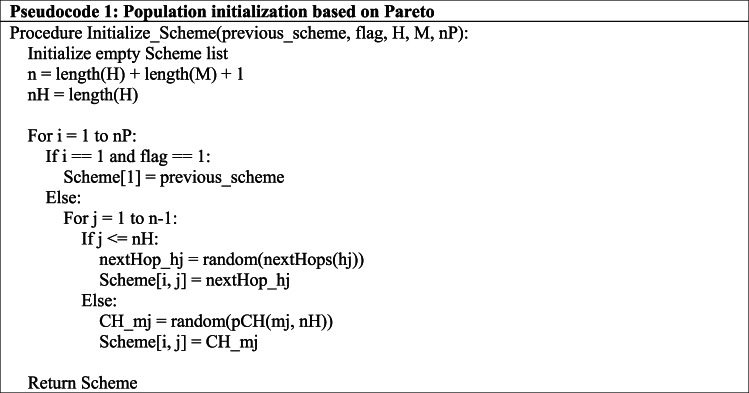


The population scheme in Algorithm 1 is initialized as an empty set in Step 2, and the numbers of every node and CH node are computed in Steps 3 and 4, respectively. The nP chromosomes are then assigned by the procedure starting in step 5. Step 8 assigns the j-th gene of the i-th chromosome in the population, unless the flag is not set to 1 (step 6), in which case step 7 assigns the starting chromosomes to previous_scheme.Give scheme (i, j) Hop_h_j. Steps 13–15 assign CH_mj to Scheme(i, j).

### Fitness function computation with Pareto

Every sensor node in a WSN has a finite amount of energy, and every node must use some energy while transmitting and receiving information from other nodes. Energy may be conserved if overall energy usage is decreased. Therefore, we must lower the overall energy usage of every node. The suggested PGAECR approach is used in this research for WSNs with permanent CHs. The CHs in this kind of WSN are in charge of gathering and sending data. Another significant issue that might impact the network life cycle is load balancing for CH_S_. If a CH has significant load-related energy consumption, the node may experience early depletion, affecting the network’s stability and energy efficiency. Changes in routing and clustered technologies may lead to changes in the CHS’s power consumption and balance of load. Under the matching clustering and routing strategy, the entire energy consumption is represented by $\:sumE$. Equation 24 demonstrates that $\:mh{E}_{ij}$ is the total of $\:sendm{h}_{Uj}$ and $\:recmh{P}_{j}$. If the sent data length is 1-bit, we may use Eq. (14) to determine $\:sendmh{E}_{ij}$.:

14 $$\:sendmhe{E}_{ij}\left(l\right)=\left\{\begin{array}{c}{E}_{elec}*l+{\epsilon\:}_{fs}*l*{d}^{2}\left({m}_{i},{h}_{j}\right),Ifd({m}_{i},{h}_{j})<{d}_{0}\\\:{E}_{elec}*l+{\epsilon\:}_{mp}*l*{d}^{4}\left({m}_{i},{h}_{j}\right),Ifd({m}_{i},{h}_{j})\ge\:{d}_{0}\end{array}\right.$$.

The energy used by h_j_ to receive 1-bit information from mi may be determined via Eq. (15):

15 $$\:recmh{E}_{ij}={E}_{elec}*l.$$

**Fig. 4:**
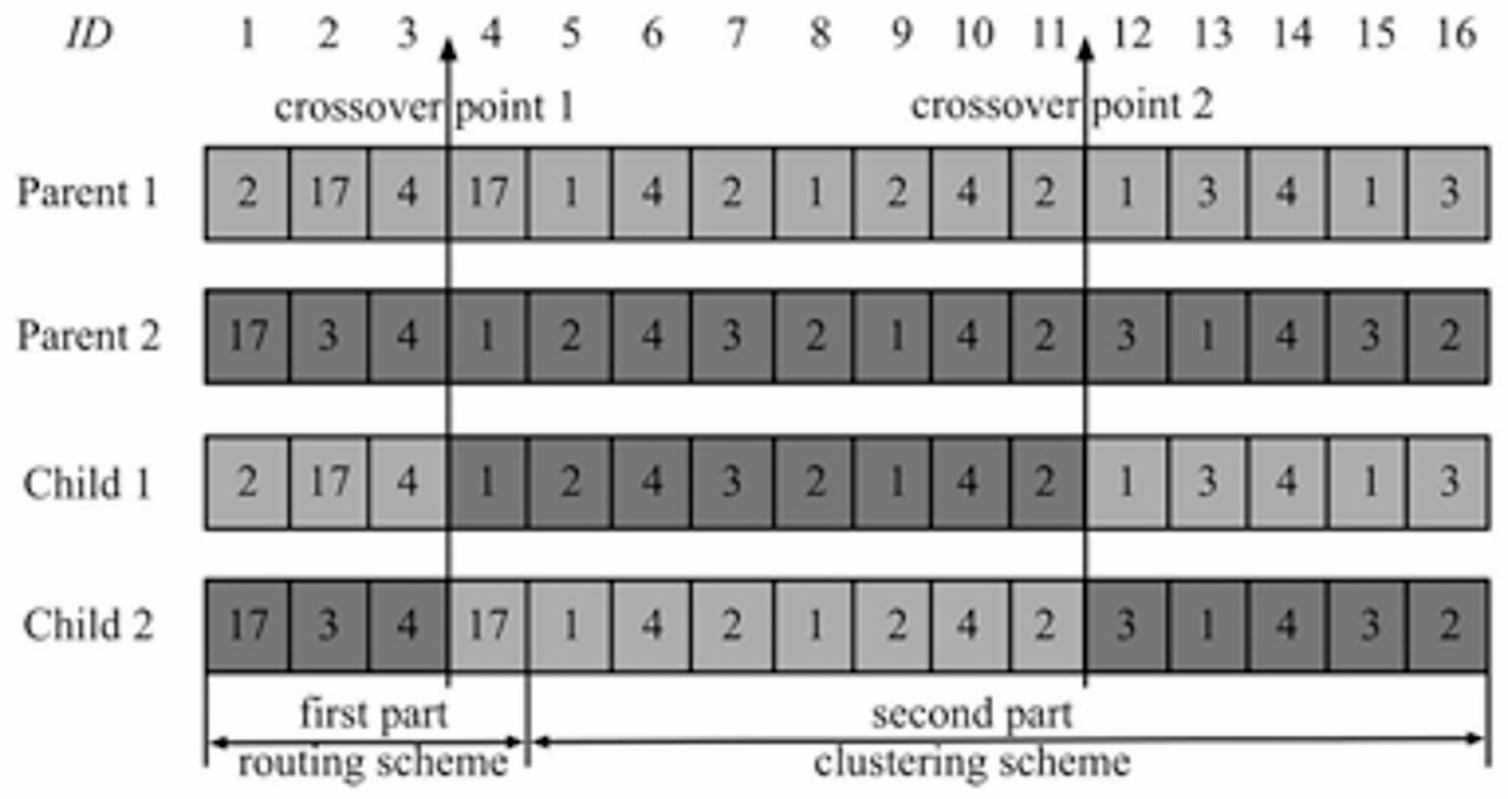
Crossover of two-parent genomes with Pareto.

If the sink node is the next hop for h$\:k$, then *È* $\:hk{h}_{\:nH+1}$ equals the energy when sending information right away to the sink nodes; if not, *È* $\:h{k}_{hT+1\:}$equals the total energy needed for interaction among all CH_S_. Since there is sufficient energy available to the node that sinks $\:{h}_{nH:1}$, the energy used by $\:{h}_{nH+1\:}$to receive information is ignored.d. Figure 4’s system chromosome determines overall energy usage under the clustering and transportation architecture. Furthermore, the data transported from CHS to their subsequent hops has a length of $\:{l}^{{\prime\:}}$- This data that is transmitted may be computed by adding up the data from the CM nodes. We must reduce the amount of resources used in every phase to extend the network’s life cycle since sensors on nodes in the network have a limited amount of energy. As a result, chromosomes with reduced energy consumption should be chosen. A chromosomal region that uses less energy tends to have a lower fitness rating. As a result, the fitness function relates to the overall energy use EI is shown in Eq. (16):

16 $$\:fit\:\propto\:sumE .$$

Next, we compute the variables associated with the CHs’ load balancing. We denote $\:Avr{g}_{i}$ as the mean residual energy allotted to every load on the CH h_i_, which may be computed as follows in Eq. (17):

17 $$\:Avr{g}_{i}=\:\frac{{E}_{resident}\:\left({h}_{i}\right)}{nL\:\left({h}_{i}\right)}.$$

Then, the mean $\:\mu\:$ of $\:vrgi$ for every CH may be determined using Eq. (18)

18 $$\:\mu\:=\:\frac{\sum\:_{i=1}^{nH}Avr{g}_{i}}{nH}\:.$$

The standard deviations of the average residual power $\:Avr{g}_{i}$, by using the values of $\:\mu\:\:$and $\:Avr{g}_{i}$based on Eq. (19) is calculated.

19 $$\:\sigma\:=\:\sqrt{\frac{{\sum\:}_{\varvec{i}=1}^{\varvec{n}\varvec{H}}(\varvec{\mu\:}-\varvec{A}\varvec{v}{\varvec{g}}_{\varvec{i}}{)}^{2}}{\varvec{n}\varvec{H}}}.$$

The system’s lifespan will be extended, and energy usage will be balanced with a smaller standard deviation $\:\sigma\:$. As a result, $\:\sigma\:$ is correlated with the fitness value based on Eqs. (20) and (21).

20 $$\:Fit\:\propto\:\:\sigma\:$$

21 $$\:\sigma\:=\:\frac{\sigma\:-\:{\sigma\:}_{min}}{{\sigma\:}_{max}-\:{\sigma\:}_{min}}.$$

$\:sumE$ and $\:sumE$ represent the greatest and lowest of $\:sumE$, whereas $\:\sigma\:max$ and $\:\sigma\:min$ denote the highest and lowest of a fitness

### Pareto genetic algorithm operation

#### Crossover and mutation using Pareto

Designing genetic code is crucial in GA. Mutation and crossover processors are among the biological operators. After a generation, the roulette wheel algorithm selects chromosomes with lower fitness. Crossover operators use these selected chromosomes to create new progeny.

Each member of the population has a single chromosomal scheme according to the suggested PGAECR methodology. As shown in Fig. 4, the chromosome is divided into two portions that provide the best route and clustering schemes. The two-point crossover technique randomly selects a crossover point in the first and second halves of both domains to enhance gene exchange. The crossover operator occurs consistently in this investigation. The first described method chooses the better-performing chromosomes from both the parent and the new offspring chromosomal. It is, therefore, possible to maintain the finest solutions in this way for the next generation. The kid chromosomes created by the crossover procedure are still functional; it should be mentioned. In the first section of the strategy, the k-th gene is chosen at random from the set $\:next$. Following crossover, the k-th gene is still part of $\:next$(h_k_). Similarly, the k-th gene is a member of the set $\:\left({m}_{k-nH}\right)$ if it is a member of the second portion of the scheme. In the GA, Mutation is employed to create better chromosomes. The suggested PGAECR approach takes advantage of the fundamental bit mutation operator. Every gene on a chromosome is subject to mutation. We presume that a mutation must occur in the k-th gene. The previous k-th allele must be replaced with a new allele chosen at random from the set $\:next$(h_k_). The preceding allele must be replaced with a new allele that is randomly chosen from the set$\:\:\left({m}_{k-nH}\right)$.

**Fig. 5:**
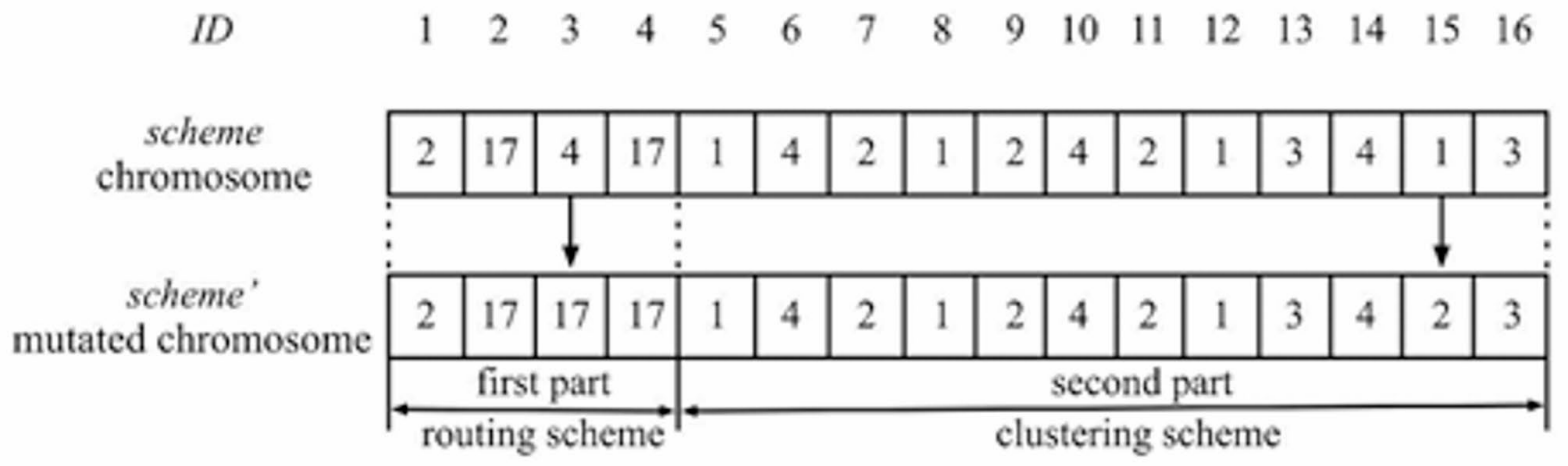
Mutation of the chromosome.

The chromosomal scheme mutation is shown in Fig. 5. The chromosome that results from a mutation is depicted in this picture. The third gene and the fifteenth gene are the two that need mutations. The initial section of the chromosome, the set $\:next$(h_3_) = *, 4,17+, contains the third gene. Subsequently, allele 4 is replaced with the number 17. Concurrently, the set (*m*11) = *1, **+** contains the fifteenth gene. Next, the prior allele 1 is replaced with the number 2.

### Pareto principle for optimization

Inspired by the way Paretoesprinciple, the Pareto Optimizing technique is a metaheuristic optimization technique. A starting group of Paretoes is created within the search area, and the objective function is used to gauge the population’s stimulus intensity. Next, a population of Paretoes A = (a1, a2…. an} is created. It begins by using the objective function to calculate the level of stimulus Ik for each Pareto. Next, iteratively calculates each Pareto’s fitness and determines the optimal location for it. In every iteration, the fitness value of each Paretoes is used to compute its scent, and a selection of numbers is produced to indicate whether to utilize the local or global searching approach based on Eq. (22).

22 $$\:{A}_{k}^{(t+1)}={A}_{k}^{t}+\left({r}^{2}*{b}_{\:}^{*}-{A}_{k}^{t}\right){F}_{k}.$$

Here, b* represents the location of the best Pareto discovered to date, r is an integer with a value between 0 and 1, F_k_ is the Paretoes k’s scent, and $\:{A}_{k}^{(t+1)}\:$is the Pareto’s present position at time t. When a global search is selected, the Pareto uses the global search equation to principle in the direction of the Pareto with the greatest fitness. If a search area is selected, the Pareto uses the location search using Eq. (23) to principle towards a spot between two other Paretoes, i and j, that it randomly picks.

23 $$\:{A}_{k}^{(t+1)}={A}_{k}^{t}+\left({r}^{2}\text{*}{A}_{i}^{t}-{A}_{k}^{t}\right){F}_{k}\:.$$

The locations of two randomly chosen Paretoes, i and j, at time t, are denoted by the variables $\:{A}_{i}^{t}$. The global search gains weight as the power exponent lowers with more repetitions. The method seems to be carried out with the process until it finds a way to stop, such when it reaches a certain amount of repetitions. At that time, it gives the best answer. Pseudocode 2 shows the Pareto Artificial Intelligence (AI) method as a second example.


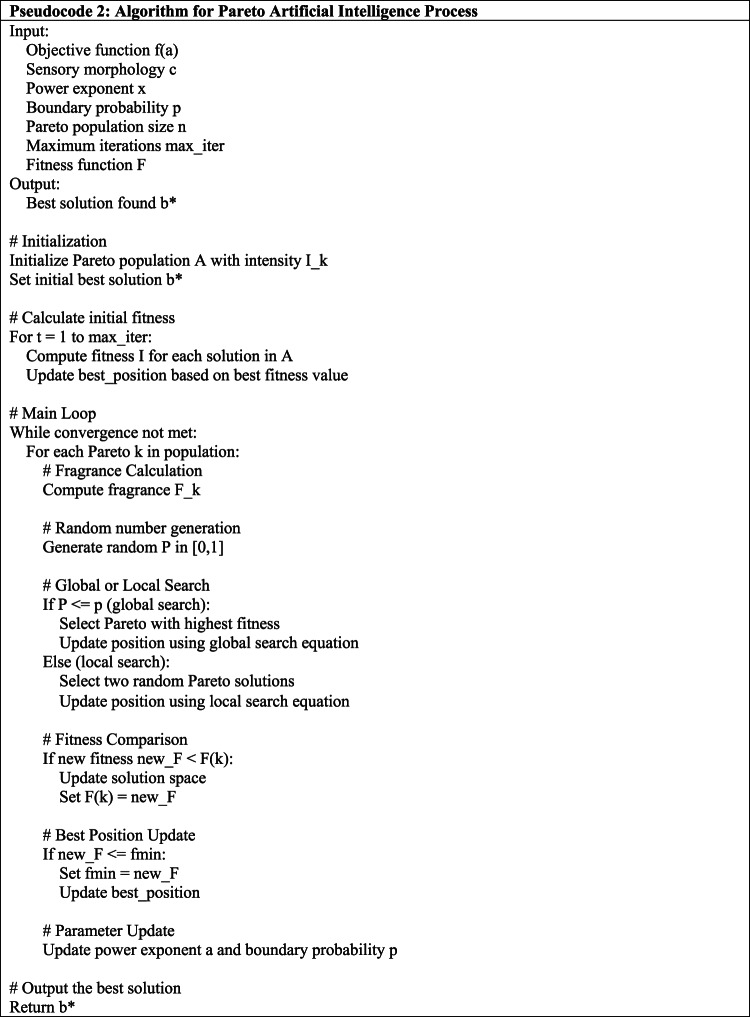


### Clustering and routing decision

In the Clustering & Routing Decision phase of the PGAECR model, optimal cluster heads and energy-efficient routing pathways are chosen. The purpose is to extend network life. Genetic algorithm solutions are used to dynamically build clusters. Cluster chiefs are selected from nodes with the most remaining energy to balance the energy load. The communication of base station is only with the nodes in cluster via the cluster heads. This is done by implementing the optimum multi-hop routing structure. Routing pathways give energy efficiency and reliability of the network top priority in a bid to limit the node overload possibilities. PGAECR extends the WSN lifetime with reduced transmission overhead and energy consumption. This is done by integrating routing and clustering into a single optimization framework.

**Fig. 6:**
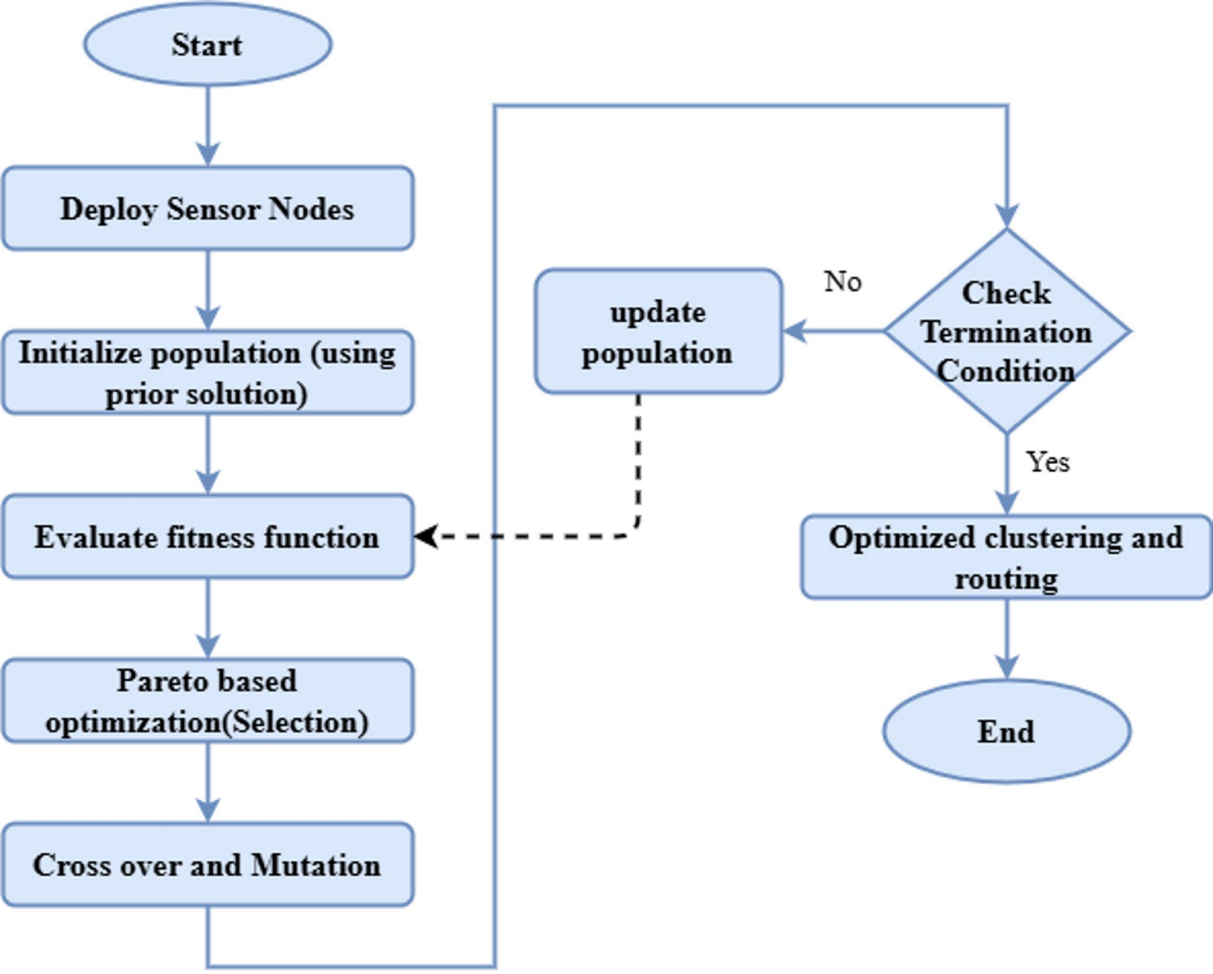
Flow chart representation of Pareto genetic algorithm operation.

The PGAECR flowchart in Fig. 6 illustrates population initialization, which integrates earlier methods for better optimization after sensor node deployment. Similarly, network duration, load balancing and energy efficiency are detailed by the fitness function. This method increases the responses by Pareto optimization-based selection, crossover and mutation. Unless a termination state has been halted, this methodology enhances the population and reaches the loop. Or otherwise, it halts with an optimal routing with the clustering solution. This systematic technique permits WSNs for the proper utility of energy and endures.

.A various-purpose extraction method is undertaken by WSN which increases the locating of clusters related on the parameters like Received Signal Power (RSP), energy exploitation, delay and route loss. The methodology improves communication along with power efficiency. Delays are wrapped along with the best node positions for steady signal communication is picked with route loss variation analysis for increasing communication. Sustainability is estimated by locating the energy patterns and relevant transmission through RSS changes analysis. Best way for translation is acquired by increasing the anchor node operations with the apt localization, maximum information and real-time adaptive methodologies for network optimization which also optimizes WSN efficiency, accuracy followed data quality. Covering more target nodes, the multi-functional derivation-based locating algorithm contains the acceptable localization efficiency. The produced GA algorithm is utilized for carrying this method as well as increases the localization results.

## Experimental results

MATLAB 2014a and NS2 simulation assessed Pareto-based Genetic Algorithm for Energy-Efficient Routing and Clustering. MATLAB is picked for its ease of prototyping, increased-level numerical computation and matrix-based data process, optimization and visualization toolboxes: a performance metric, fitness and extended genetic algorithm framework. NS2 has formulated the WSN node communication, signal propagation, energy transfer and routing dynamics. NS2, an entirely efficient network research discrete event simulator efficiently model the transmit delays, packet streams and protocol stacks. Communication and energy efficiency of the given strategy are correctly located while undergoing the process. MATLAB for algorithm development and NS2 for network simulation have made the energy utilization of PGAECR, network lifeduration and routing efficiency to be estimated in varied surroundings.

The strategy is relevant over the WSN Dataset for best group and power routing. It uses the NS2 and simulated data sets presented in MATLAB instead. Simulations define area, node as well as communication and energy methodologies. The analysis is conducted using MATLAB 2014a on an Intel(R) Core(TM) i5-3317U CPU and 6144 MB RAM PC. The algorithm is contrasted with CGAL, DV-HOP, CENTA and EDV-HOP, an evolutionarily distant vector-hop. In a real network, 200 powered node sensors have been randomly placed, 25% of these being anchor nodes and the remainder references or unknowns. Tables 3 and 4 list this study’s experimental parameters.

**Table 3:** Parameters access in sensor field and GAS.

Simulator factor	Range
Overall No. of vertex	200
Deploy area field	200*200 m^2^
Range of communication	40 m
No. of Anchor	51
No. of Unanchor	101
Max iteration	301
No. of cluster	6
Size of population	51
Chromosome length	151
No. of gen	0.6
Rate of mutation	0.9

**Table 4:** Factors used in the source model.

Factors	Range
Origin energy	2 J
D_0_	88 m
Size of packet	200 bits
E_T_	50 nJ/bit
E_R_	50 nJ/bit
E_D_	50 nJ/bit/signal
Power amp in free space energy consumption	10 pJ/bit/m^2^
Power amp in multipath fading	0.013 pJ/bit/m^4^

### Statistical validation using ANOVA

An ANOVA test can be performed to check if there is a statistically significant improvement in routing efficiency and clustering performance. The test evaluates several algorithms, including PGAECR, GA, PSO, and classic heuristic approaches, by comparing their mean performance metrics. These metrics may include execution time, energy efficiency, and clustering accuracy. There is an assumption of no significant difference between the algorithms in the null hypothesis (H₀), but at least one approach is suggested to perform much better in the alternative hypothesis (H₁).

The statistical significance of the improvements in PGAECR can be confirmed if the ANOVA test produces a p-value less than 0.05. To further narrow down the possible algorithm pairings with statistically significant differences, a post-hoc Tukey test can be run. This statistical validation adds weight to the argument that the suggested method is preferable to more conventional methods and confirms its dependability.

**Fig. 7:**
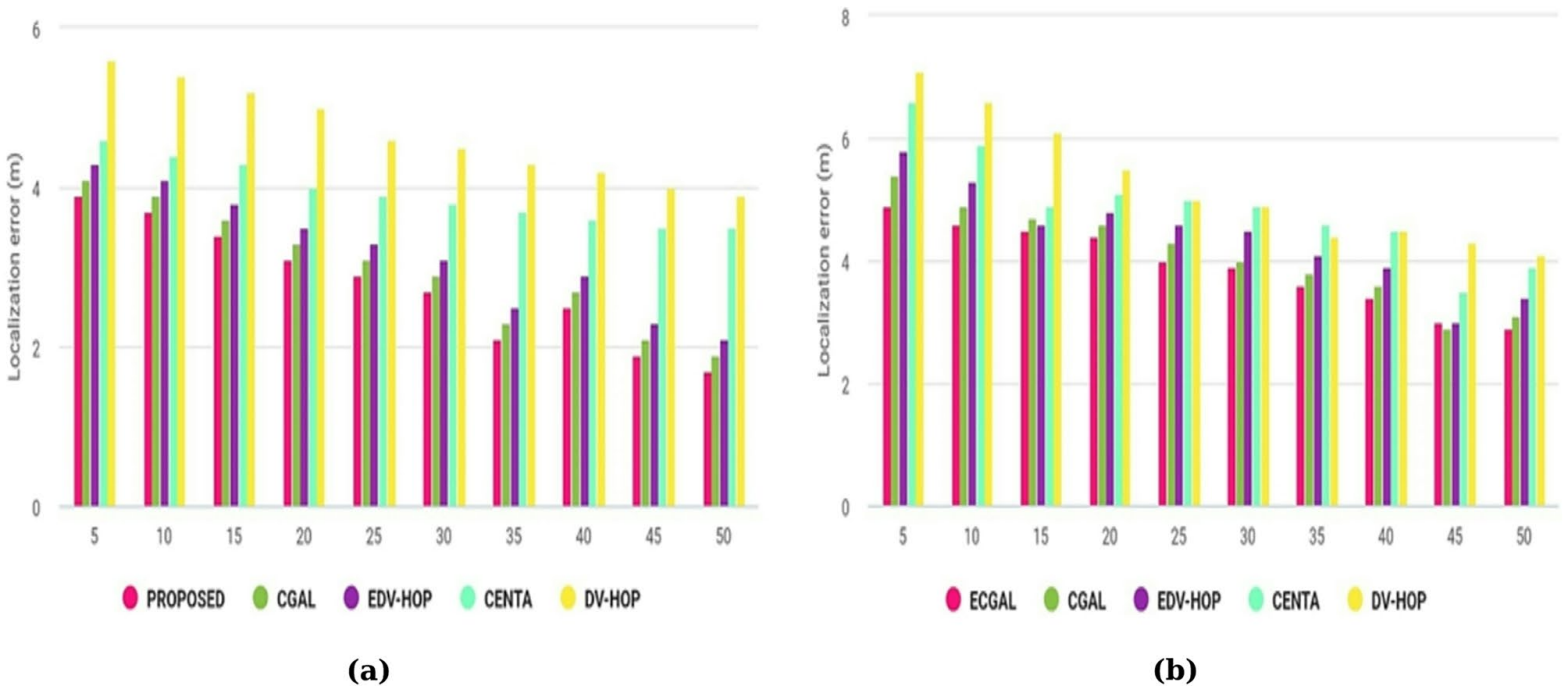
Distortion in localization relative to the number of anchor nodes and transmission area.

A Figure 7a illustrates that the novel method outperforms conventional location-based methods in terms of error location, which supports this conclusion. Nearly every technique used works reliably with the same setup. The extra anchoring nodes in the network provided the target nodes with additional reference points, which allowed the proposed model to decline smoothly. On the other hand, when there are enough anchoring nodes, the network is strengthened because the separation between the unknown vertices and the anchoring nodes increases closer. CGALS, EDV-HOPS, and CENTAs also showed reduced localization errors compared to the model output. Figure 7b shows continuous simulated seconds rise when the transmission range extends. To assess the effectiveness of our methods, the transmission distance concurrently begins at 5 m and increases by 5 m over time.

When the propagation radius slopes, the node location precision increases, and the localization error is minimized. Ultimately, the proposed achieves improved precise location outcomes as the signal’s range grows.

**Fig. 8:**
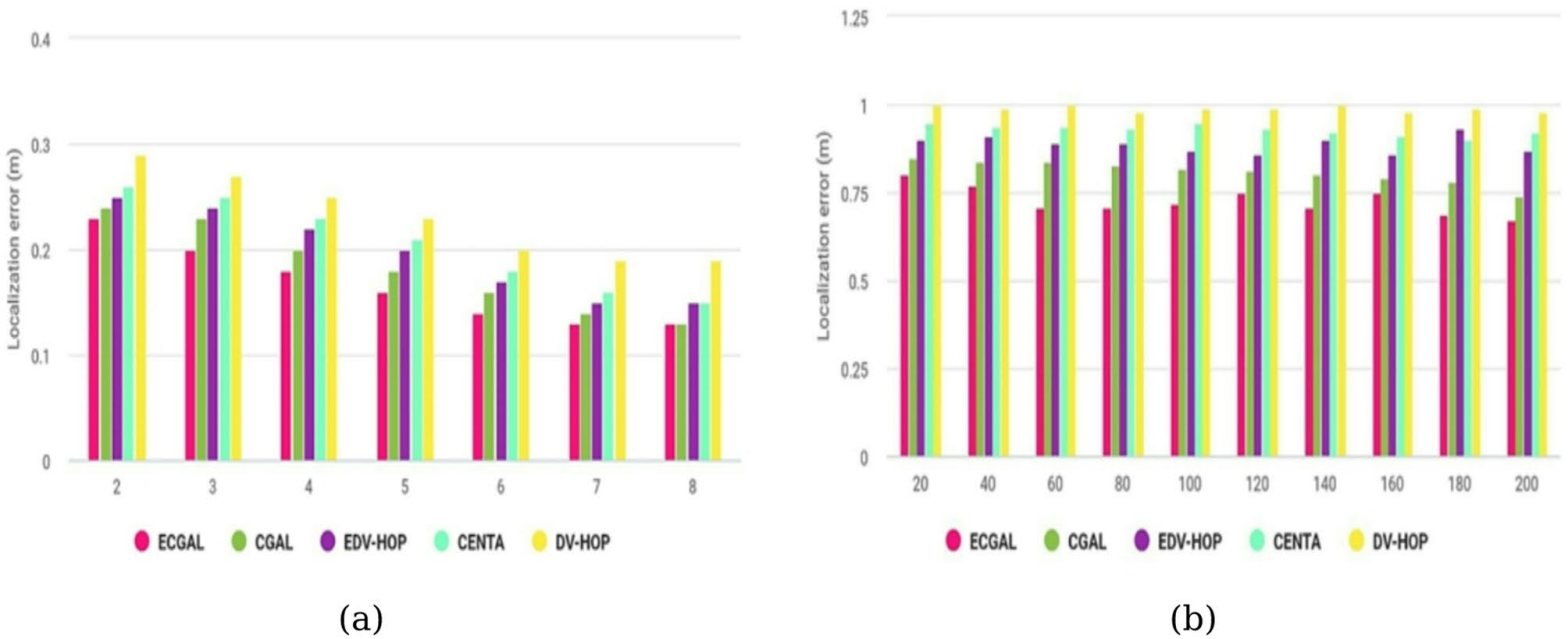
Localization inaccuracy concerning the overall node count and number of groups.

Figure 8a displays the experimental findings for localization error calculated against different node counts. Moreover, the positioning error for each technique begins to decrease as the quantity of active sensor nodes rises. The proposed technique has the lowest localization error score of all our localization methods. More points of reference are discovered as the number of clusters approaches 200, which aids in the node’s more accurate localization. However, as the number of powered nodes increases, so do the lifetime contributory variables in DVHOP and CENTA. Figure 8b displays the localization error versus the number of groups for each of the various algorithms considered in this paper. The method of clustering increases the system’s energy conservation. As the quantity of groups increases, the localization error decreases. However, CENTA performs almost similarly to EDV-HOP due to its unique clustering capacities. In DV-HOPS, the transfer scale has sufficient excited nodes, meaning that more node locations can be discovered for every cluster.

**Fig. 9:**
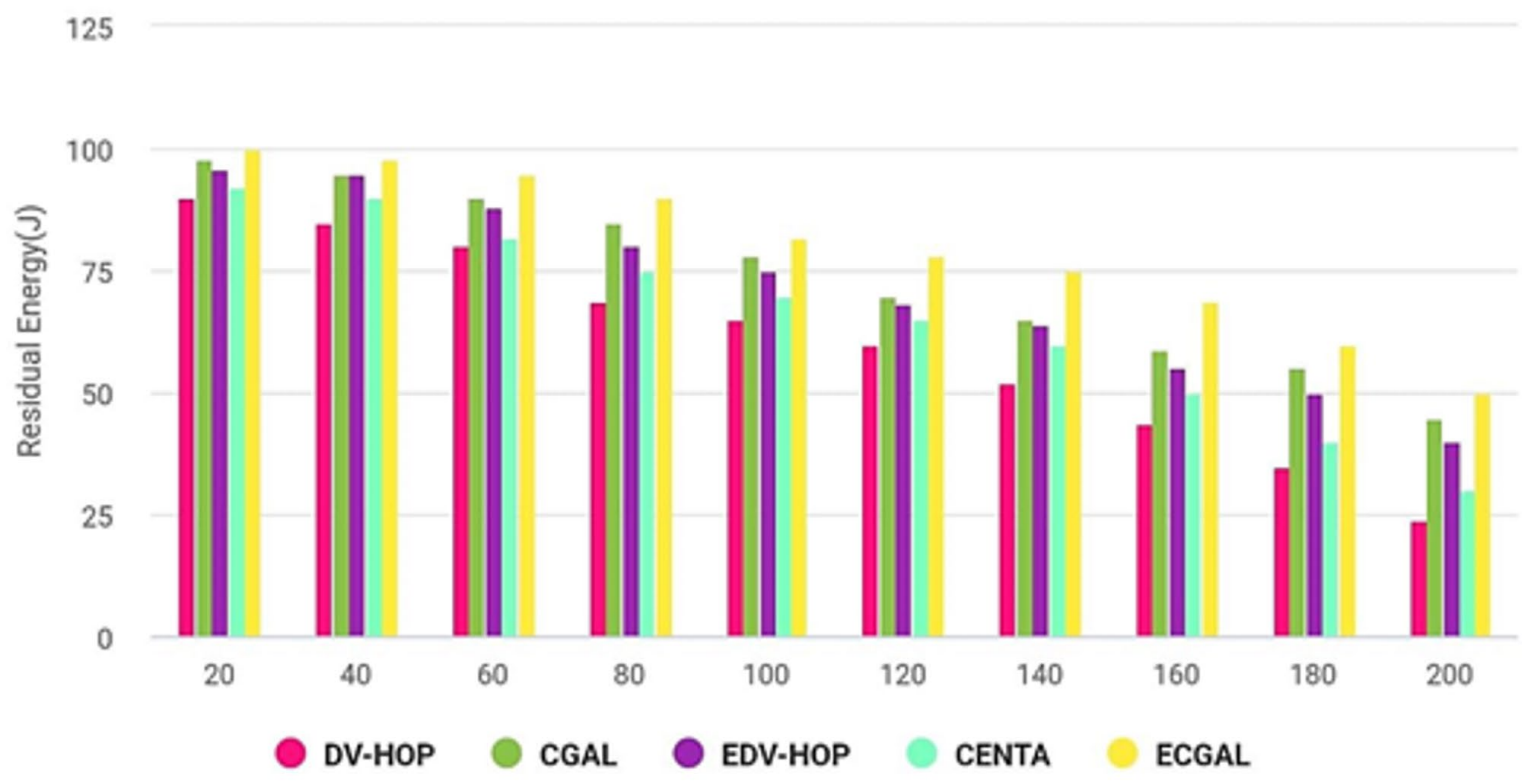
Residual energy.

The residual energy is shown versus the number of repetitions in Fig. 9. In contrast to CGALS, EDV-HOPS, CENTAS, and DV-HOPS, PROPOSED offers significant energy reductions. Every technique started to decline more and more until it reached 80 repetitions, at which point it began to decrease dramatically. The energy remaining after 120 repetitions is around 70 J, so PROPOSED finds more residual energy than CGAL. This might be because the cluster centers were optimally elected, and the distance that separated the intracluster and intercluster was equal. Both CGAL and EVD-HOP function identically at the starting point. Our graph indicates that after 160 repetitions, the energy left in CENTA and EDV-HOP was 50 J and 55 J, respectively. Lastly, a decrease in residual energy shortens the network’s lifespan, raising the number of control packets (overhead) exchanged.

**Fig. 10:**
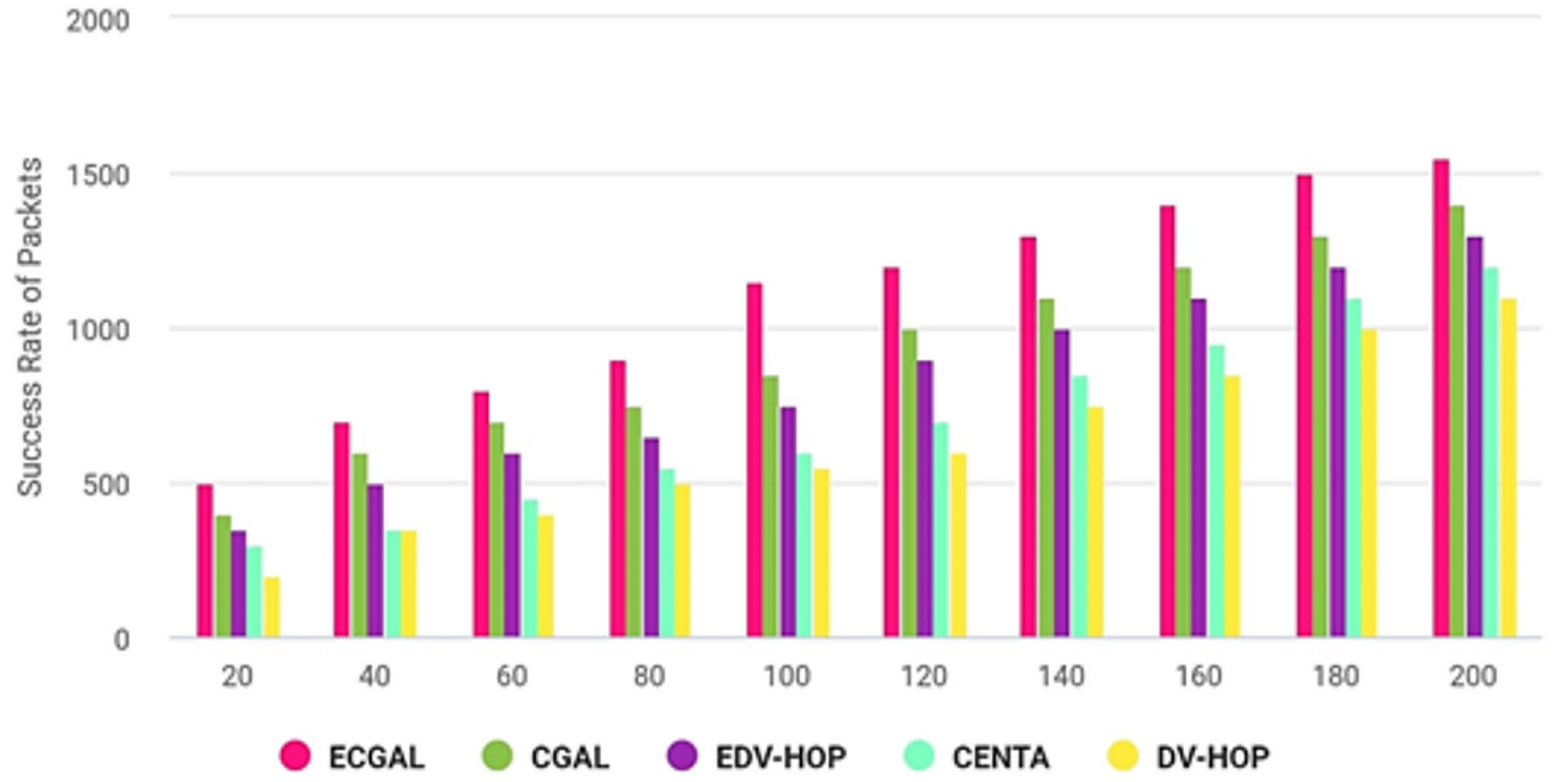
Transmission rate.

The algorithm’s processor period is calculated for 200 repetitions over seconds in Fig. 10. The overall success rate of the packets delivered is defined as the ratio of the number of communications created to the number of communications conveyed to the end destination. Last, concerning convergence rate, PROPOSED outperforms CGALS, EDV-HOPS, CENTAS, and DV-HOPS. The proposed method successfully carries 90% of the data to its intended location. CGAL and EDV-HOP demonstrated greater efficiency than CENTA and DV-HOP as repetitions increased.

**Fig. 11:**
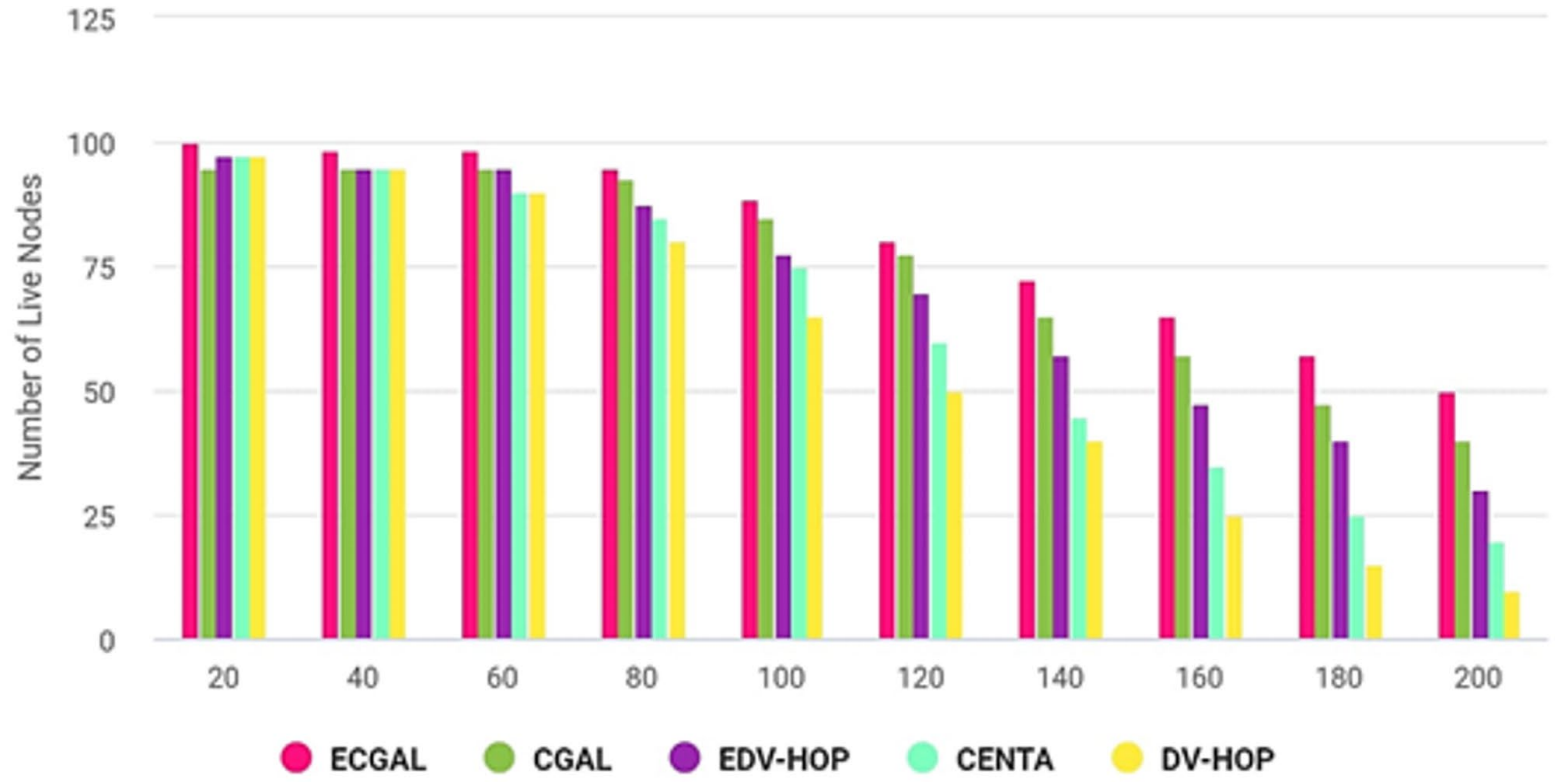
Network lifespan.

The network’s lifespan is illustrated in Fig. 11 and compared to the EDV-HOPS, CENTAS, and DV-HOPS approaches. CGALS and the proposed methodology significantly extend the network’s lifespan. The evaluation of the live nodes is conducted by raising the iteration count to 200. The network’s electrified sensor nodes eventually lose energy after a few repetitions. At 180 iterations, PROPOSED has 60 active nodes compared to CGAL’s 50. In the meantime, there are only around 40 active nodes in EDV-HOP and 25 in CENTA. The figure’s active nodes, indicated by dashed lines, suggest that the approach outlives other approaches. Due to its energy-efficient cluster localization strategy, which utilizes a genetic algorithm, PROPOSED outperformed CGALS, EDV-HOPS, CENTAS, and DV-HOP algorithms.

**Fig. 12:**
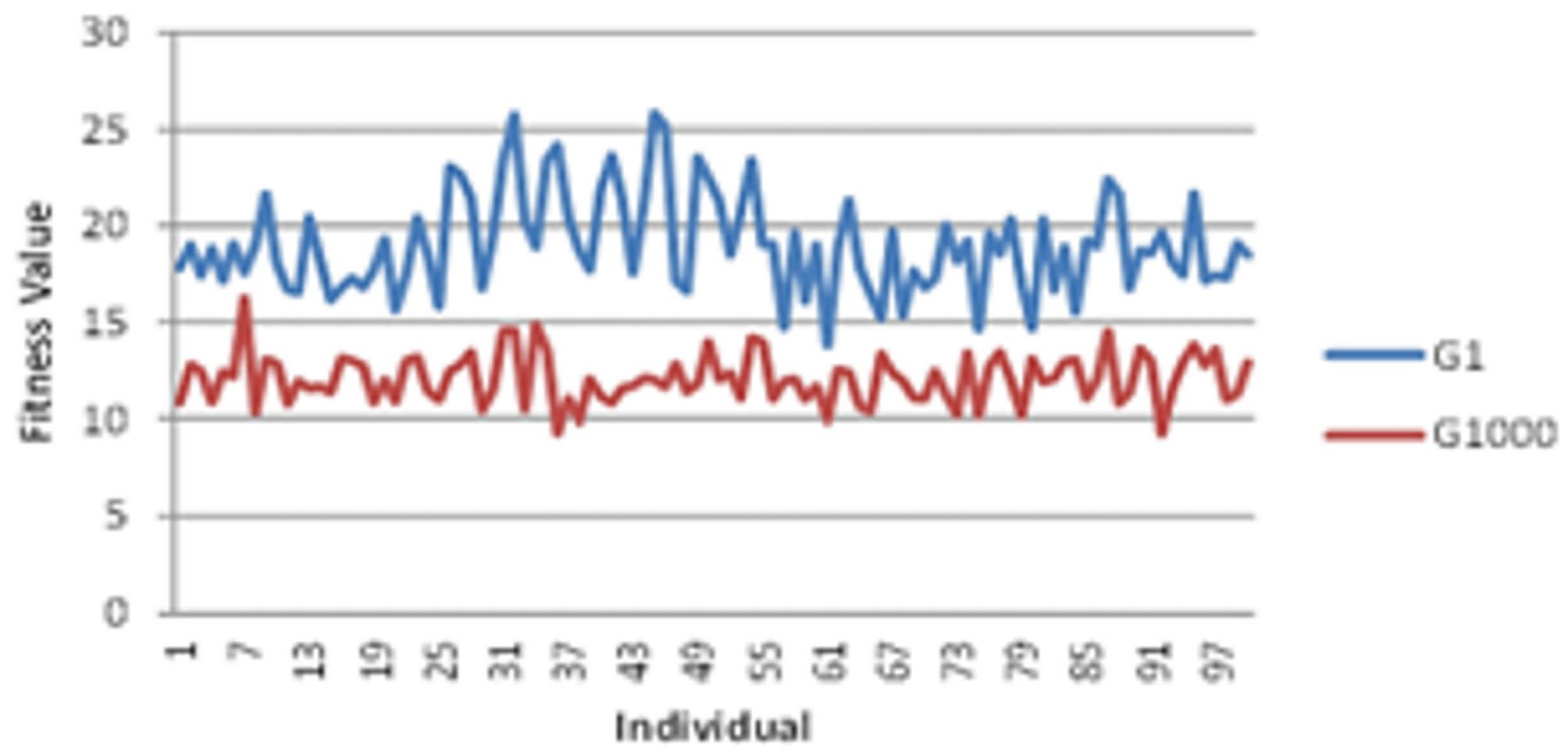
Average fitness value.

Compared to the first iteration, the fitness value determines the optimal value. The effect levels for each objective function were changed based on the importance of the subject matter. By examining F’s behavior, it can be seen that its values were higher in the first generation than in the last, which suggests that the process of creating future generations was focused on enhancing the genes to be as good as possible in the final generation. These are the metrics used. Key performance indicators are optimization technique efficiencies. Overall Energy - OE represents the general energy the network or its sensor nodes spend in a certain period or operational round. CE - Cluster Energy represents the amount of energy the cluster heads use, as these nodes have to aggregate data from other nodes inside their respective clusters. OCE: Optimized Cluster Energy, which is the energy consumed by the cluster heads after applying optimization algorithms. This can lead to an improvement in energy savings. SORE: The sum of Residual Energy is the total residual energy within all the sensor nodes after completing a certain number of operations. It gives a view of the network’s sustainability over time. At the same time, F stands for Fitness Value, which is the overall performance of the network during its use in various optimization algorithms, specifically regarding the fulfillment of criteria related to energy efficiency and load balancing. The genetic makeup of F in larger variations in the first generation but less fluctuation in the final generation, indicating consistency in the last generation’s alleles. To investigate this topic further (gene stability across several iterations), the fitness function F_in_ is shown in Fig. 12.

**Fig. 13:**
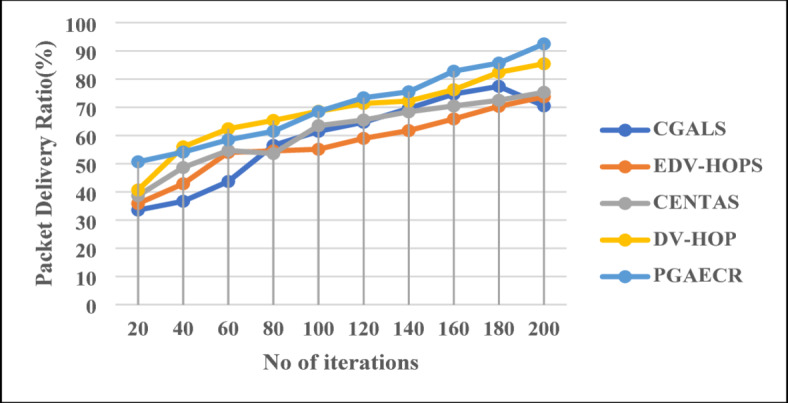
Packet delivery ratio.

The number of successfully delivered data packets from the source to destination points out the PDR of a network. This test evaluates the reliability and data transmission of the routing protocol. The proposedis better than CGALS, EDV-HOPS, CENTAS and DV-HOP in clustering and routing energy. The technique functioned was proof. The suggested method achieves a 92.4% packet delivery ratio (PDR), which is more significant than the others, as shown in Fig. 13. This enhances data reliability, reduces packet loss, and improves network performance. Pareto-based genetic optimization selects optimal cluster heads and paths for this development. This improvement reduces transmission failures. The graph below shows PDR percentages for each procedure:

PGAECR is a new Genetic Algorithm that extends the lifetime of Wireless Sensor Networks (WSNs) and improves energy efficiency. It is based on the Pareto principle. Combining the best findings from prior networking sessions into subsequent iterations enhances the efficacy of the search. The method uses a single chromosome to encode routing and clustering, and its fitness function considers overall energy expenditure and load balance. By improving load balancing, decreasing energy consumption by 12.4%, and increasing network lifetime by 15.7%, experimental data demonstrate that PGAECR outperforms all existing methods.

The evolutionary tasks, including selection, crossover, mutation, and fitness evaluation, have the greatest impact on the computing complexity of PGAECR. N is the population size, G is the generation number, and L is the chromosomal length. Overall, the complexity is O(NL) every generation due to the fitness evaluation’s O(L) time operation, which evaluates the performance of routing and clustering. Due to its impact on genes, selection—which is frequently accomplished by tournament or roulette-wheel methods—contributes to O(N log N), whereas mutation and crossover add O(NL). As a result, as the size of the issue increases, the overall complexity per generation is O(NL + N log N), and the worst-case complexity for G generations is O(GN²). The competitive scalability that PGAECR maintains makes it suitable for mid-to large-scale optimization problems, in contrast to classical heuristic techniques that operate in O(N log N) or O(N²) and other metaheuristic approaches with complexities ranging from O(GN²) to O(GN³).

**Table 5:** Comparative summary.

Metric	PGAECR	ReLeC-MO [23]	OptiGeA [26]	BWO-Harmony^28^
First Node Die (FND) (Rounds)	78	62	69	60
Half Nodes Die (HND) (Rounds)	138	115	126	108
LND (Rounds)	192	165	178	153
Energy Left (at 120)	70 J	58 J	60 J	53 J
Alive Nodes (at 150)	72	55	61	50

 These results graphically represents that PGAECR is usable in practice as it performs better and lasts longer in a greater variety of WSN settings. (Table 5). The experimental findings demonstrate that the proposed Pareto-based Genetic Algorithm for Energy-Efficient Clustering and Routing (PGAECR) consistently outperforms other approaches, such as CGAL, EDV-HOP, CENTA, and DV-HOP, on all key performance metrics. PGAECR maintains more energy in the network, makes it last longer, delivers more packets, finds locations more accurately, and balances the load better among cluster heads. These benefits result from using a single chromosome for routing and clustering, combining the best historical solutions for more rapid convergence, and a multi-objective fitness function that balances energy efficiency with load distribution. Briefly, PGAECR is an appropriate method for lifespan increase and the efficiency improvement of WSNs.

## Conclusion

This research introduces a resilient and energy-efficient clustering and routing methodology for Wireless Sensor Networks (WSNs) with an innovative Pareto-based Genetic Algorithm (PGAECR). The suggested strategy is more effective than previous ones, as it addresses issues such as inconsistent energy use, limited network lifespan, and hazardous routing decisions. PGAECR differs from traditional algorithms in that it employs multi-objective Pareto optimization and historical learning. It allows for the application of the best solutions from prior rounds again. This improves both the speed of convergence and the quality of the solution. PGAECR simplifies the optimization process by consolidating all clustering and routing choices into a single chromosome. It also ensures that route construction and cluster head selection work in tandem effectively. Its health role includes both total energy use and load balancing measures. This ensures that energy is distributed evenly and lowers the chance of early node failures. Simulation findings show that PGAECR uses less energy, sends more packets, improves localization accuracy, and significantly extends the network’s lifetime compared to benchmark algorithms such as CGAL, EDV-HOP, CENTA, and DV-HOP. PGAECR is an excellent choice for large and dynamically changing WSN networks since it is adaptive and tolerant. This is the optimal response to the real-world scenarios where power saving and maintaining the network operational for an extended time is extremely critical. To further enhance it in reality, numerous researches have been formulated for seeing its performance with the actual-time computing data as well ass hardware limitations.

## Data Availability

The datasets generated and analysed during the current study are available from the corresponding author on reasonable request.
